# Detection and Quantification of Tire Particles in Sediments Using a Combination of Simultaneous Thermal Analysis, Fourier Transform Infra-Red, and Parallel Factor Analysis

**DOI:** 10.3390/ijerph16183444

**Published:** 2019-09-17

**Authors:** Demmelash Mengistu, Vegard Nilsen, Arve Heistad, Knut Kvaal

**Affiliations:** 1 Faculty of Science and Technology, Norwegian University of Life Sciences, 1430 Ås, Norway; vegard.nilsen@nmbu.no (V.N.); arve.heistad@nmbu.no (A.H.); knut.kvaal@nmbu.no (K.K.); 2 The Municipality of Ås, Myrveien 16, 1430 Ås, Norway

**Keywords:** detection, quantification, PARAFAC, FTIR, microplastics, tire particles, sediment, method

## Abstract

Detection and quantification of tread wear particles in the environment have been a challenge owing to lack of a robust method. This study investigated the applicability of a combination of Simultaneous Thermal Analysis (STA), Fourier Transform Infra-Red (FTIR), and Parallel Factor Analysis (PARAFAC) in the detection and quantification of tire particles from formulated sediments. FTIR spectral data were obtained by heating 20 samples in STA. Among the 20 samples, 12 were tire granules in formulated sediments (TGIS) containing 1%, 2%, 5%, and 10% by mass of tire granules, while the remaining eight contained 0.5, 1, 2.5, and 5 mg of tire granules only (TGO). The PARAFAC models decomposed the trilinear data into three components. Tire rubber materials in tire granules (RM) and a combination of water and carbon dioxide were the components identified in all samples. The linear regression analysis of score values from the PARAFAC models showed that the RM quantity predicted were comparable to measured values in both TGIS and TGO. Decomposing the overlying components in the spectral data into different components, and predicting unknown quantity in both sample types, the method proves robust in identifying and quantifying tire particles from sediments.

## 1. Introduction

Tread wear particles has become an environmental concern because of their significant global microplastics contribution [1,2]. Vehicle tires release about 11.5% of mass during their service life [3,4]. The considerable amount of particles released to the environment is composed of many chemicals, including natural and synthetic rubber polymers, fillers, reinforcing agents, processing aids, accelerators and retarders, adhesives and activators [5]. Tread wear particles generated during driving by rolling shear of tread against pavement [6] picks more substances from the road surface and other traffic related emissions [1]. These particles are deposited along roadsides with wildly varying quantity. Parts of the tread wear particles enter the marine environment [7] and spread even to the remote areas [2]. Tread wear particles are recognized as threat to human health and marine and terrestrial organisms. Adverse health and environmental effects from airborne particulate matters of tire wear are reported in [1,8] while other studies (e.g., [2]) included potential adverse effects from terrestrial and aquatic environment. However, quantification of tread wear particles and the associated risks remains unanswered [2]. In fact, quantification is a common challenge for emerging pollutants such as graphene nanoparticles [9].

The failure to detect and accurately quantify tread wear particles arises from lack of a robust method to detect tire particles in the environment [1,6]. Methods applied to detect microplastics in the environment or analytical methods established for single polymer particle detection are not suitable for identification of tread wear particles [1,6], because the use of these methods requires a challenging extraction step and extensive pretreatment [10]. Work on indirect methods of detection through markers could not establish dependable markers from additives in the tire making process [6]. However, studies have reported that component analysis methods are reliable to detect polymers in the environment [11,12]. Rubber material, which is the core component in tires [13], therefore, makes a good candidate for component analysis to determine the presence and to estimate the quantity of tread wear particles in sediments and soils [6]. Simultaneous Thermal Analysis (STA) or application of Thermal Gravimetric Analysis (TGA) and Differential Scanning Calorimetry (DSC), are standard tools in compositional analysis of multi-component materials like tire [14,15]. Simultaneous Thermal Analysis, besides its comparatively lesser time and sample preparation demands, gives information on two physical processes, mass change and heat flow, on the same sample simultaneously [16]. However, physical properties alone are not sufficient in identifying chemical components [16]. Coupling the STA to Fourier Transform Infra-Red (FTIR) spectroscopy provides additional and powerful information about the chemical properties of materials [17,18]. Nevertheless, direct application of this method, on complex matrices like sediments and soils where the quantity of tread wear particles is expected to be low, is unreliable [6]. One factor limiting the effectiveness of the method is the generation of overlying components in the TGA [14] which gives non-conclusive FTIR matches referenced to the usually pure compound in the library [19]. Quantitative analysis through calibration curve is also impossible because of the overlying components [11].

Mathematical tensor decomposition methods can resolve FTIR spectra with overlying components. Parallel Factor Analysis (PARAFAC) has shown great versatility in this respect in resolving the multi-component matrix into underlying analytes [20] and offers a reliable foundation to estimate quantity of the components based on Beer-Lambert’s law [21,22]. The method requires tri-linearity—the same number of components underlie the chemical variation in each dimension of the dataset; variability—no identical signals or co-varying intensities between two chemical components; and additivity—the total signal is due to the linear superposition of a fixed number of components [22]. Choosing the right number of components for the model and acquisition of appropriate signal-to-noise ratio of spectral data is critical for the analysis [23].

The objective of this study is to introduce a new method for detecting and estimating quantity of rubber materials from formulated sediment (RM) for possible application in the detection and quantification of tread wear particles from soil and sediment.

## 2. Materials and Methods

### 2.1. Tire Granules

Three different sizes of tire granules (<1.2 mm, 1–2.8 mm, 2.5–4 mm) were obtained from Ragn-Sells, a producer and distributor of tire granules by grinding discarded tires in Norway and Sweden [24]. The source of the granules is coded as plastic and rubber (1912 04) in the European waste code. The product specification states that the tire granules contain 58% elastomer designated as Styrene-Butadiene Rubber (SBR). Styrene butadiene rubber (SBR) is a synthetic rubber. However, it represents all rubber materials in tires in the manufacturers’ context. The SBR content of the tire granules is estimated with similar method to the International Federation of Association of Football (FIFA) test Method 11, Thermal Gravimetric Analysis [25]. The producer’s specification was not verified by independent study and the rubber material in tire granules (RM) is relatively high compared to 40–50% rubber materials in tires estimated by [26,27,28]. However, we used the stated proportion to represent all RM in this experiment.

### 2.2. Formulated Sediment

To ensure experiment repeatability and knowledge of particle size and content in the samples, sediment was formulated according to OECD guidelines [29] for the testing of chemicals with composition of 5% organic matter, 75% quartz sand, and 20% kaolinite clay. Finally, water-equivalent to 50% by volume of the formulated sediment was added to the mix [29]. Organic matter used in the sediment preparation is a conifer bark (EAN: 7058782362833) obtained from a plant shop (Plantasjen). Quartz sand (0.2–0.4 mm) and kaolinite clay (CAS: 1332-58-7) were obtained from Radasand and VWR respectively.

### 2.3. Sample Preparation

We prepared two types of samples for the experiment (Table 1) using Mettler Toledo XP205 DeltaRange analytical balance. The first sample type was tire granules in formulated sediment (TGIS). We prepared TGIS by placing a 1000 mg of formulated sediment in four small beakers of size 20 mL each. A total of 10, 20, 50, and 100 mg of finely ground tire granules (<1.2 mm was selected for this experiment [24]) were added to the four beakers in random order giving four (1%, 2%, 5%, and 10%) rubber material concentrations named RM1, RM2, RM3, RM4. We shook each beaker by hand for a proper mix. We then collected 50 mg of TGIS from each beaker and placed in separate aluminum crucibles in three replicates. Based on a 58% ratio, the collected 50 mg samples formed four classes of rubber materials in tire granules (RM) equivalent to 0.29 mg in 1% TGIS, 0.58 mg in 2% TGIS, 1.45 mg in 5%TGIS, and 2.9 mg in 10% TGIS. The second sample type contained tire granules only (TGO). We used TGO to test if the detection and quantification of RM from TGIS and TGO is comparable. We prepared TGO samples by placing 0.5, 1, 2.5, and 5 mg of tire granules in separate aluminum crucibles in two replicates. The second sample type corresponded to the rubber material quantities in TGIS. The samples were assigned identification numbers. Total sample was limited to 20 because of the high cost of data generation and limited budget. Two replicates were enough for TGO since homogeneity is assumed.

### 2.4. Experimental Flow and Procedure

**Figure 1:**
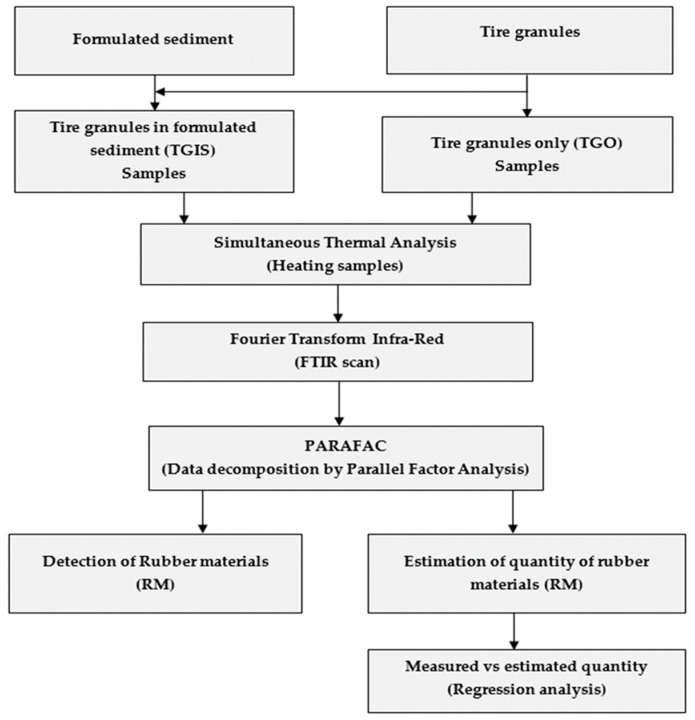
Graphical presentation of flow of the experiment (sample preparation and sampling, sample heating, Fourier Transform Infra-Red (FTIR) scanning, data decomposition, rubber material detection (RM), estimation of quantity of rubber materials (RM), and regression analysis).

Figure 1 presents the flow of the experiment.

Simultaneous Thermal Analysis: The experiment was conducted at the Norwegian Institute of Bioeconomy Research (NIBIO)—a laboratory which is primarily set for the analysis of wood and wood products. We placed crucibles with samples on automatic sample changer and fed to the Simultaneous Thermal Analyzer-STA 449 F1 Jupiter (Netzsch, Selb, Germany), type S, for heating according to pre-programmed arrangement. The heating process was programmed to progress at the rate of 10 °C/min temperature rise from 4 to 800 °C in a N_2_ (20 mL/min) protective gas environment. Evolved gases were purged to the attached FTIR instrument by N_2_ gas (50 mL/min) every 2.28 °C increase on average. See [30] for more information on STA. Weight loss of samples were registered in percentage as the heating progressed. We converted the weight loss to mg per temperature ramp by multiplying original sample mass with percentage difference between consecutive temperature ramps.

Fourier Transform Infra-Red (FTIR) (Bruker, Billerica, MA, USA): We used Bruker TENSOR 27 with external gas cell FTIR to scan the spectra of the evolved gases. The average signal of 16 scans of FTIR spectra in the wavenumber range of 600–4000 cm^−1^ was collected with a resolution of 1.9 cm^−1^ during the heating process.

### 2.5. Data and the PARAFAC Model

We obtained two sets of data per sample from STA and FTIR instruments. The STA data included temperature ramp and percentage mass loss. The FTIR data contained wavenumber cm^−1^ and absorbance intensity. The first task was to assign temperature ramp from the STA data to the FTIR data. The data were handled using MATLAB (The MathWorks Inc., R2018a, Natick, MA, USA). PARAFAC models were built using Partial least squares (PLS) Toolbox for MATLAB version 8.6; Eigenvector Research Inc. (Wenatchee, WA, USA) [31].

FTIR data from each sample was represented by a matrix (Wavenumber cm^−1^ × Temperature °C), representing spectral wavenumbers and heating ramp along their dimensions. As all FTIR data were measured using a similar temperature ramp, individual matrices could be stacked to form a three-way data structure of Samples (S), wave numbers (W), and temperature ramps (T). This gave a trilinear data of 12 and eight samples × 1672 wavenumber cm^−1^ × 333 temperature steps (spectra), making it suitable for a multiway analysis [20].

Two PARAFAC models were applied to the trilinear data to decompose into sets of scores and loadings, a procedure similar to [23,32]. Each data point in the dataset (X_SWT_) can then be described according to the [22] equation (Equation (1))

(1) $$X_{SWT}=\overset{F}{\underset{f=1}{\sum }}a_{Sf}b_{Wf}c_{Tf}+e_{SWT},$$

where: *S* = 1–12 in TGIS, and 1–8 in TGO, *W* = 1–1672, *T* = 1–333; *f* represents a PARAFAC component and each component has 12 and 8 *a*-values (scores); one for each sample. Each component has 1672 *b*-values; one for each wavenumber and 333 *c*-values; one for each temperature ramp. $e_{SWT}$ is the residual, representing the variability not accounted for by the model. Several number of possible components (2, 3, 4, 8) were tested and the best number of components to use was selected based on model diagnostics; namely residuals, the core consistency and fit values. See [33] for information on core consistency and model fit. A preprocessing of multiway centering along mode 2 was used in TGO and no preprocessing was needed in TGIS.

PARAFAC was preferred because it can find pure spectra from overlying components and residuals if the right component number is used in the model [23]. Mode loadings of the PARAFAC model offer reliable information because *b* and *c* in Equation (1) are scaled estimates of the component at specific FTIR spectrum and temperature respectively.

### 2.6. Rubber Material Identification

Loadings of the PARAFAC model carry fingerprint information about the substances under evaluation. Wavenumber cm^−1^ and temperature loadings of a component were evaluated against the fingerprint markers of rubber constituents (natural rubber and synthetic rubber) of tire (Table 2 and Table 3) for detection of the presence of RM in TGIS and TGO.

### 2.7. Quantity Estimation

Quantity of RM can be estimated from PARAFAC scores which is represented by $a_{Sf}$ in Equation (1) [22]. We calibrated the score values of TGIS and TGO samples against measured RM using PLS linear regression to assess the estimation performance. The regression was used in reverse to predict the quantity from score values of the PARAFAC model. The root mean square error of cross-validation (RMSECV) was calculated by Equation (2) [20].

(2) $$RMSECV=\sqrt{\frac{\sum_{l=1}^{L}{\left(yl-\hat{y}l\right)}^{2}}{L}},$$

where *yl =* predicted tire quantity of the *l*th sample not included in the calibration; and $\hat{y}l$ = known tire quantity of the lth sample. *L* = number of sample.

## 3. Results

### 3.1. Sample Weight Loss in Simultaneous Thermal Analysis (STA)

**Figure 2:**
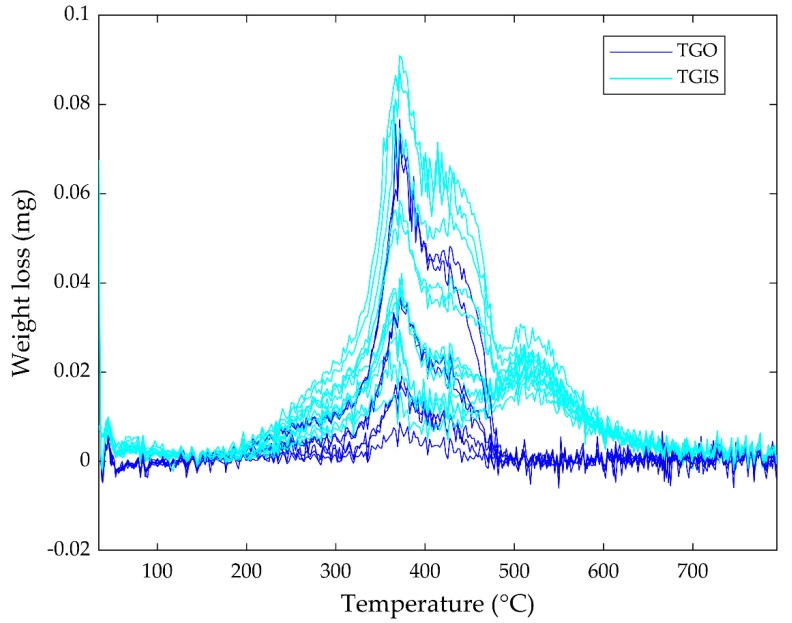
Weight loss (mg) of all 20 samples during heating in the Simultaneous Thermal Analysis (STA) instrument. TGO = Tire granules only, TGIS = Tire granules in formulated sediment.

Gases evolved from samples because of degradation during heating in the STA. Purging the evolved gases to FTIR created weight loss in all samples. The weight loss as a function of temperature is presented in Figure 2. Weight loss is observed at temperature range of 200−650 °C with peak loss rate around 370, 420, and 510 °C in tire granules in formulated sediment (TGIS) samples. The weight loss shows a narrow range (200–490 °C) with peak in loss rate around 370 and 420 °C in tire granules only (TGO) samples. The area under the weight loss curves and the peaks of the curves are higher in higher rubber materials in tire granules (RM) quantity classes indicating increased degradation with increased RM quantity. The wider temperature range and the higher degradation intensity in TGIS samples compared to the TGO samples shows generation of additional gases from organic substances in TGIS. All weight loss curves show jagged structure indicating existence of noise in the data.

### 3.2. Landscape Surfaces of FTIR Data

**Figure 3:**
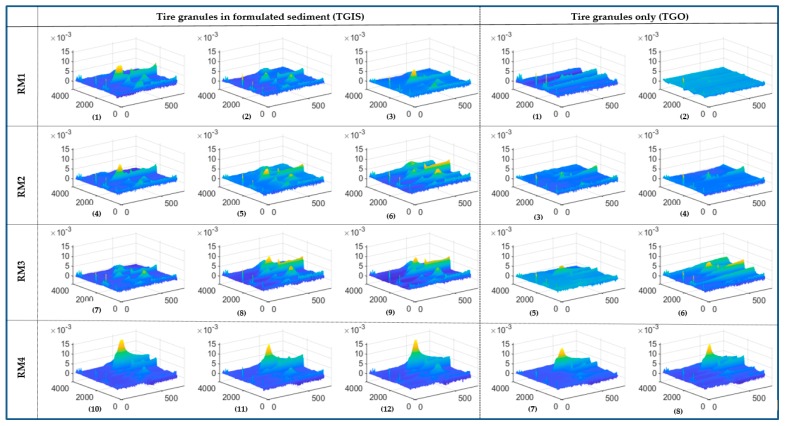
Landscape surfaces of the 20 raw FTIR spectral data. The first three columns in the figure are spectral data of samples in tire granules in formulated sediments (TGIS). The last two columns represent samples in tire granules only (TGO). The RM amount remain similar within rows and increase along the columns. The X-axis represent wavenumber (0−4000 cm^−1^), Y-axis represent temperature (0−800 °C), and z-axis represent intensity of the spectra (absorbance).

The FTIR data of evolved gases were assessed for intuitive understanding of the underlying components of the samples. Landscape surfaces of 20 FTIR data from TGIS and TGO are presented in Figure 3. Samples presented in each row have equal amount of tire granules (see Table 1). However, the landscapes show variation between the first three samples in TGIS and the next two samples in TGO containing equal amount of tire granules. The variation in landscape surfaces is detectable visually in the lower quantity profiles. Spectral intensity increases in all samples (except number 7) with increased amount of RM. Multiple peaks are observed in all of the landscape surfaces of the FTIR data.

### 3.3. The PARAFAC Model and Results

**Figure 4:**
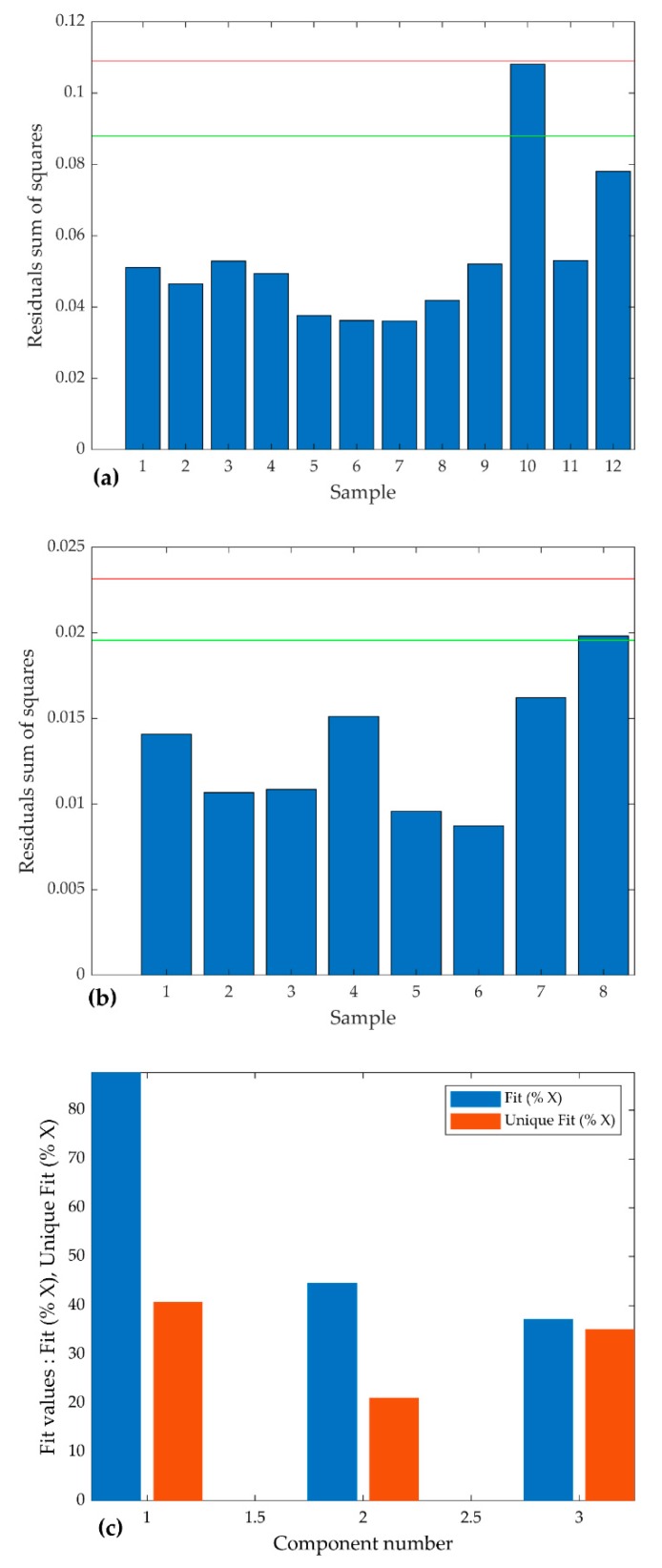

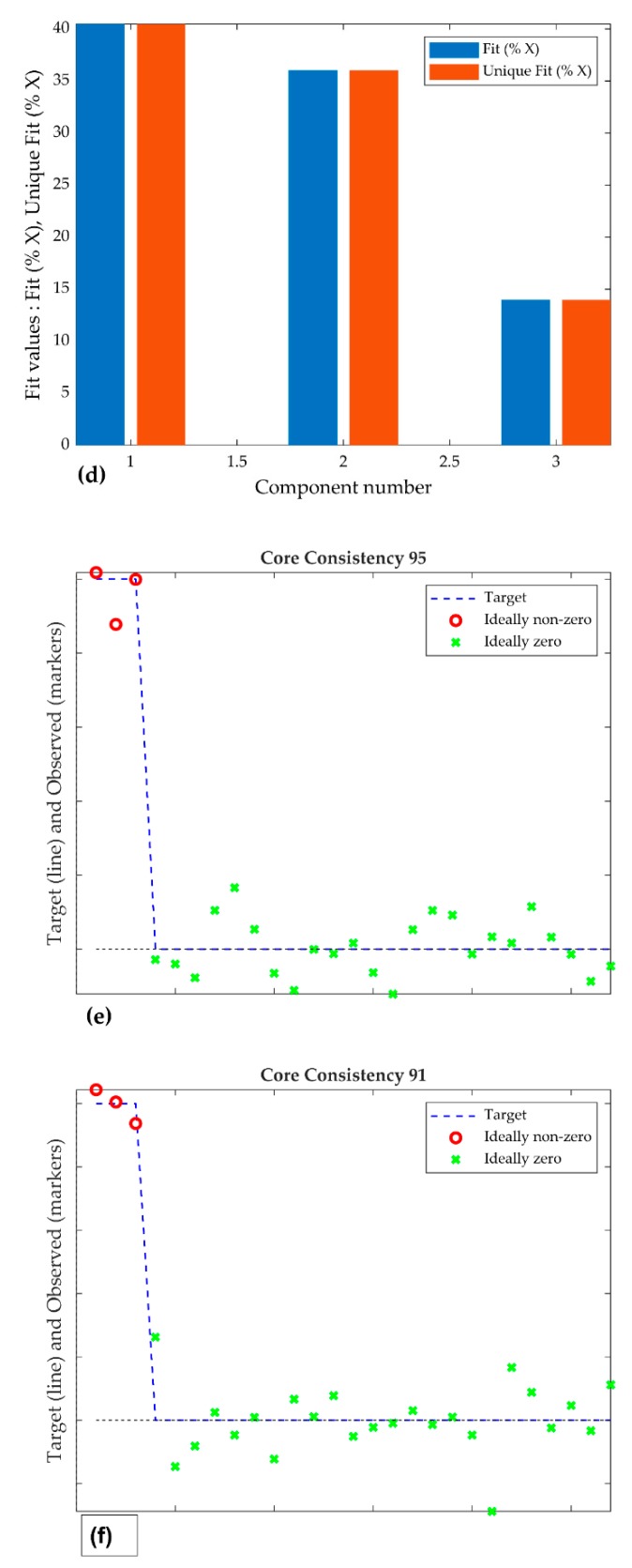
Diagnostic of the Parallel Factor Analysis (PARAFAC) models. (**a**) Residuals sum of squares of TGIS, (**b**) residuals sum of squares of TGO, (**c**) model fit and unique fit of component in TGIS, (**d**) model fit and unique fit of components in TGO, (**e**) core consistency of TGIS, and (**f**) core consistency of TGO.

Following several runs using different number of components, PARAFAC models with three components explained most of the variation in the data and thus deemed appropriate for our data to decompose the FTIR spectral data of both TGIS and TGO. The models captured 89.5% and 90% of explained variations in TGIS and TGO respectively. Low residuals in both sample types, except for sample number 10 in TGIS where a higher value is observed (Figure 4a,b). No variation is observed between model fit (the percentage of variations explained by each component) and unique fit (model fit of a component that is not shared by other components) in TGO (Figure 4d) while variation is observed between model fit and unique fit in component 2 and 3 in TGIS (Figure 4c). The models attained a very high core consistency, indicating a super diagonal core tensor G (Figure 4e,f). See [31] for information on core consistency and model fit.

**Figure 5:**
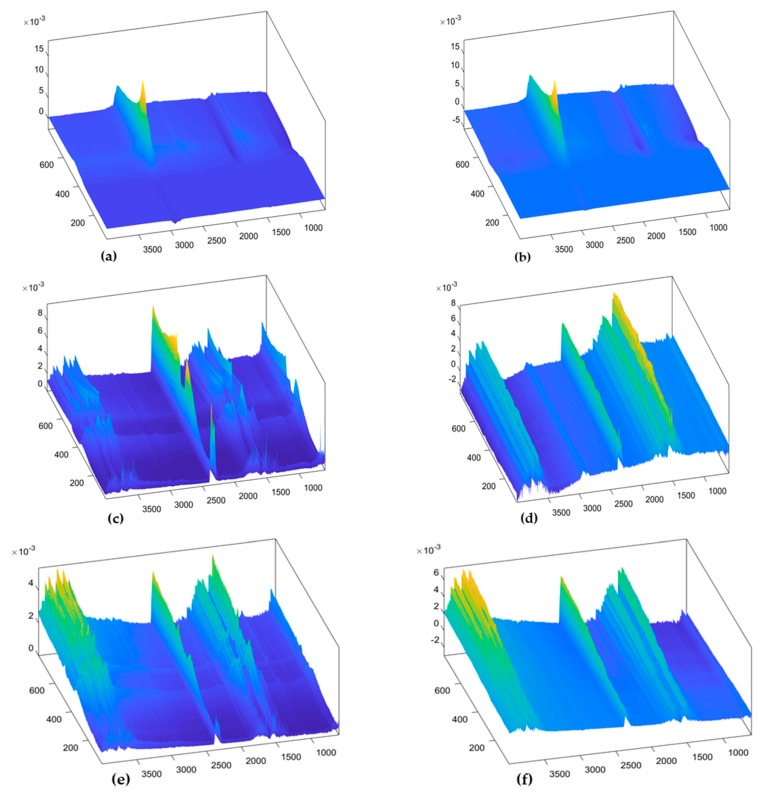
Loading surfaces of the three components of the PARAFAC models. (**a**) Component 1 of TGIS, (**b**) component 1 of TGO, (**c**) component 2 of TGIS, (**d**) component 2 of TGO, (**e**) component 3 of TGIS, and (**f**) component 3 of TGO. X-axis indicates wavenumber in cm^−1^, Y-axis indicates temperature in °C, Z-axis indicates component value.

The models with three components better exposed the differences between the components visibly, either in structure or in values of the components. Landscape surfaces of the three components are presented in Figure 5 in a rearranged order than how they appeared in the respective models. The surfaces display distinct appearance in structure from the landscape surfaces of the raw data.

Landscape surfaces of the three components from the PARAFAC model of TGIS corresponds to landscape surfaces of the components of TGO, except component 2 where the appearance looks different (Figure 5c,d). Landscape surfaces of component 1 in TGIS and TGO (Figure 5a,b) display a similar structure with a structure in the raw data that gained increased visibility with increase in RM.

Two loadings and a score occurred in each component in both PARAFAC models (Equation (1)). The wavenumber cm^−1^ loadings are expressed by mode 2, and the temperature loadings are expressed by mode 3 while scores are expressed by mode 1.

**Figure 6:**
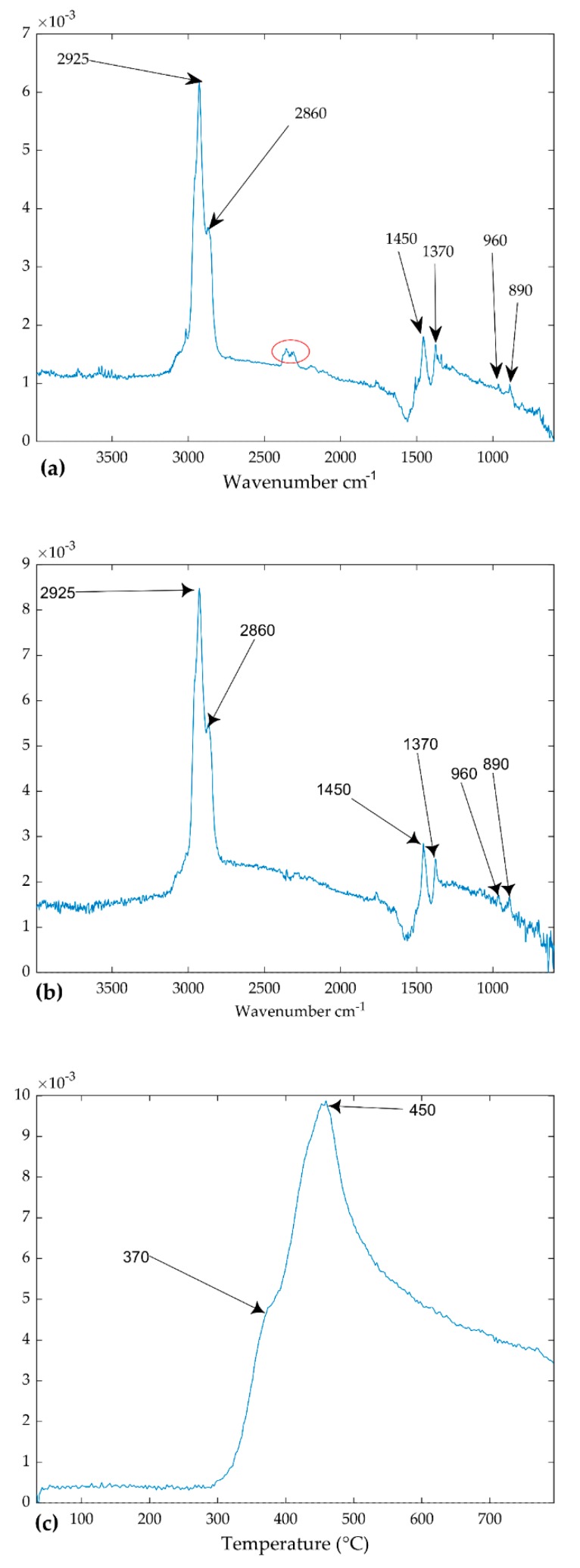

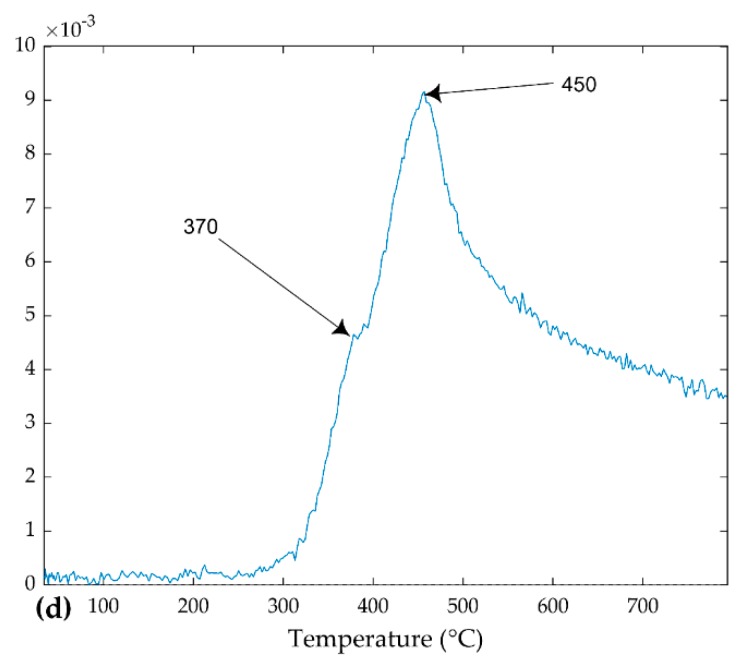
Loadings of the PARAFAC model. (**a**) Mode 2 loadings of component 1 (TGIS), (**b**) mode 2 loadings of component 1 (TGO), (**c**) mode 3 loadings of component 1 (TGIS), and (**d**) mode 3 loadings of component 1 (TGO). Y-values are scaled values of the components. The red circle in (**a**) shows a peak that is not seen in (**b**).

**Figure 7:**
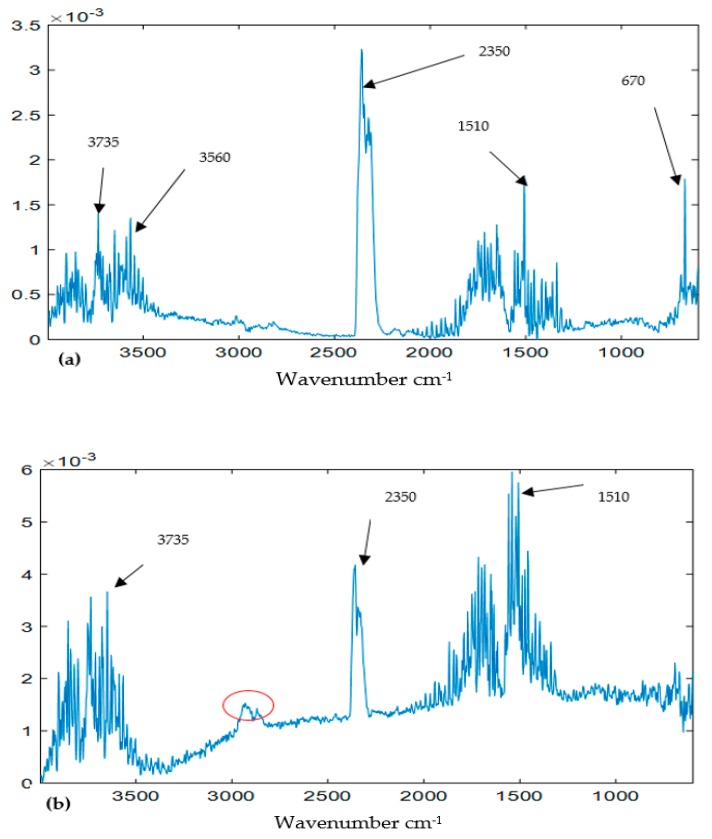

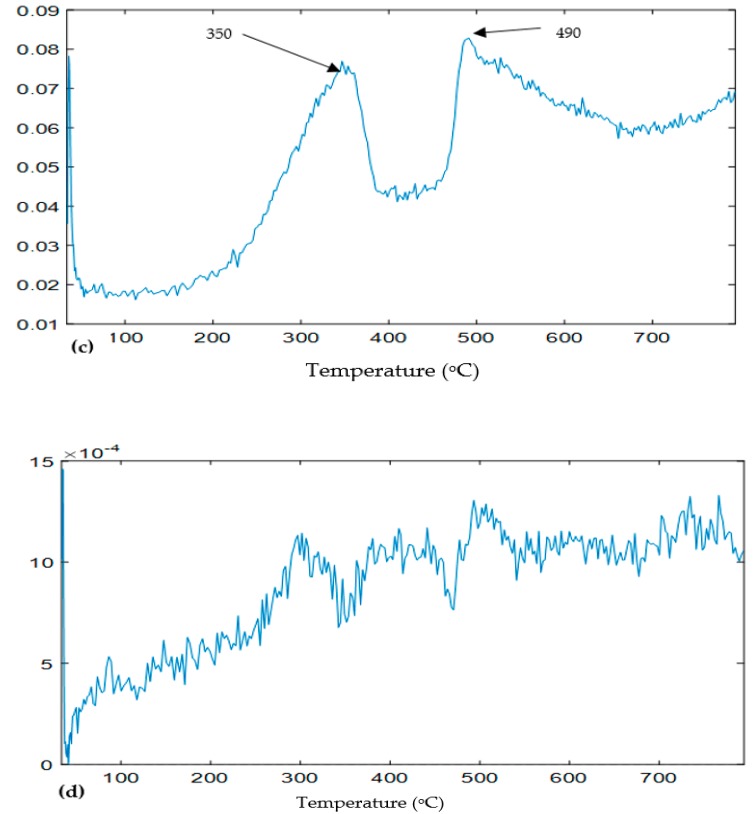
Loadings of the PARAFAC model. (**a**) Mode 2 loadings of component 2 (TGIS), (**b**) mode 2 loadings of component 2 (TGO), (**c**) mode 3 loadings of component 2 (TGIS), and (**d**) mode 3 loadings of component 2 (TGO). X-values are wavenumber cm^−1^ in (**a**,**b**) and temperature °C in (**c**,**d**). Y-values are scaled values of the components. The red circle in (**b**) shows a peak not seen in (**a**).

**Figure 8:**
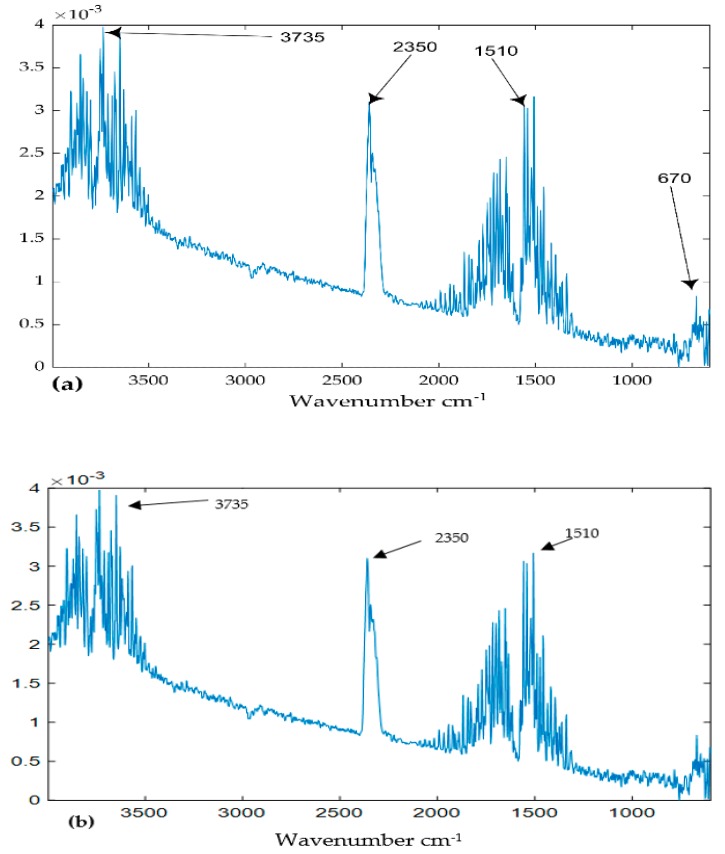

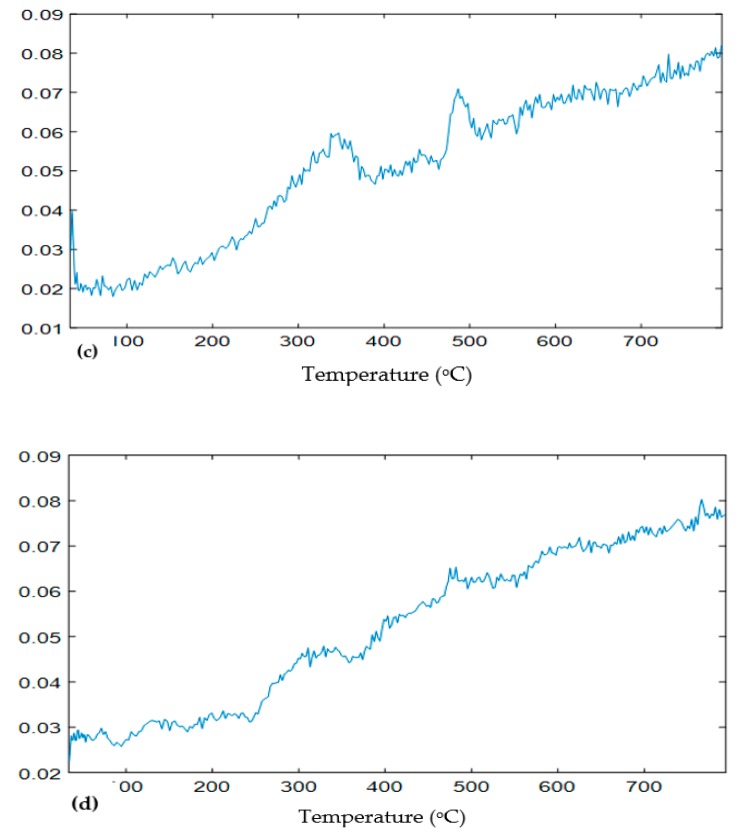
Loadings of the PARAFAC model. (**a**) Mode 2 loadings of component 3 (TGIS), (**b**) mode 2 loadings of component 3 (TGO), (**c**) mode 3 loadings of component 3 (TGIS), and (**d**) mode 3 loadings of component 3 (TGO). X-values are wavenumber cm^−1^ in (**a**,**b**) and temperature °C in (**c**,**d**). Y-values are scaled values of the components.

Wavenumber cm^−1^ loadings: Wavenumber cm^−1^ reveals the chemical properties of components as it carries fingerprint information about the substances under evaluation. Figure 6a,b show the wavenumber cm^−1^ of component 1 represented by mode 2 loadings of the models of TGIS and TGO respectively. The two loadings exhibit similar peaks around 2950, 2853, 1450, 1370, 960, and 890 cm^−1^. The wavenumber cm^−1^ loadings of TGIS shows a peak around 2350 cm^−1^ (red circle in Figure 6a) that was not seen in TGO loadings. The wavenumber cm^−1^ loadings of component 2 of TGIS and TGO show peaks at around 3735, 3560, 2356, 1510, and 670 (Figure 7a,b). The wavenumber cm^−1^ loadings of component 2 of TGO shows a peak around 2900 cm^−1^ (red circle in Figure 7b) that was not seen in TGIS loadings. Wavenumber loadings of component 3 of both TGIS and TGO exhibit similar peaks with component 2 wavenumber cm^−1^ loadings (Figure 8a,b).

Temperature (°C) loadings: Mode 3 loadings show temperature in °C at which the components are generated during heating of the samples. Temperature loadings of component 1 start rising at a temperature around 300 °C and reaches maximum at a temperature around 450 °C in both TGIS and TGO (Figure 6c,d). The loadings of component 2 of TGIS, peaks at a temperature around 350 and 490 °C (Figure 7c). Temperature loadings of component 2 of TGO increases with temperature (Figure 7d). Component 3, which have similar wavenumber cm^−1^ loadings with component 2, shows continuously increasing temperature loadings in both TGIS and TGO (Figure 8c,d). Temperature loadings peaks observed in component 1 in both TGIS and TGO correspond to the maximum weight loss in the STA instrument (Figure 2). Component 2 in TGIS also showed maximum peaks in the range where maximum weight loss was observed.

**Figure 9:**
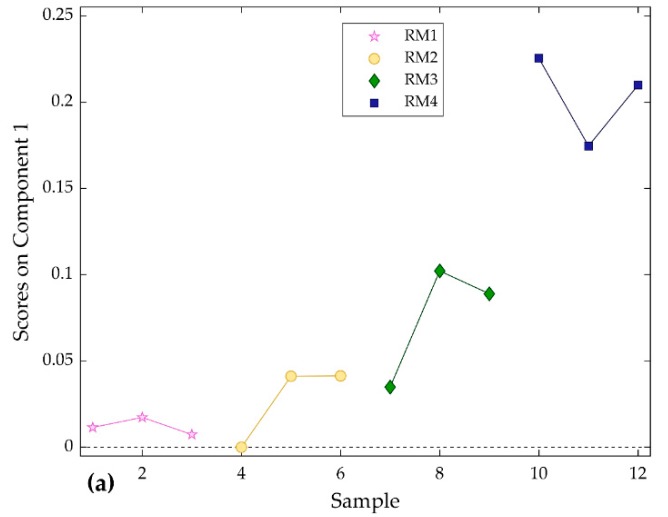

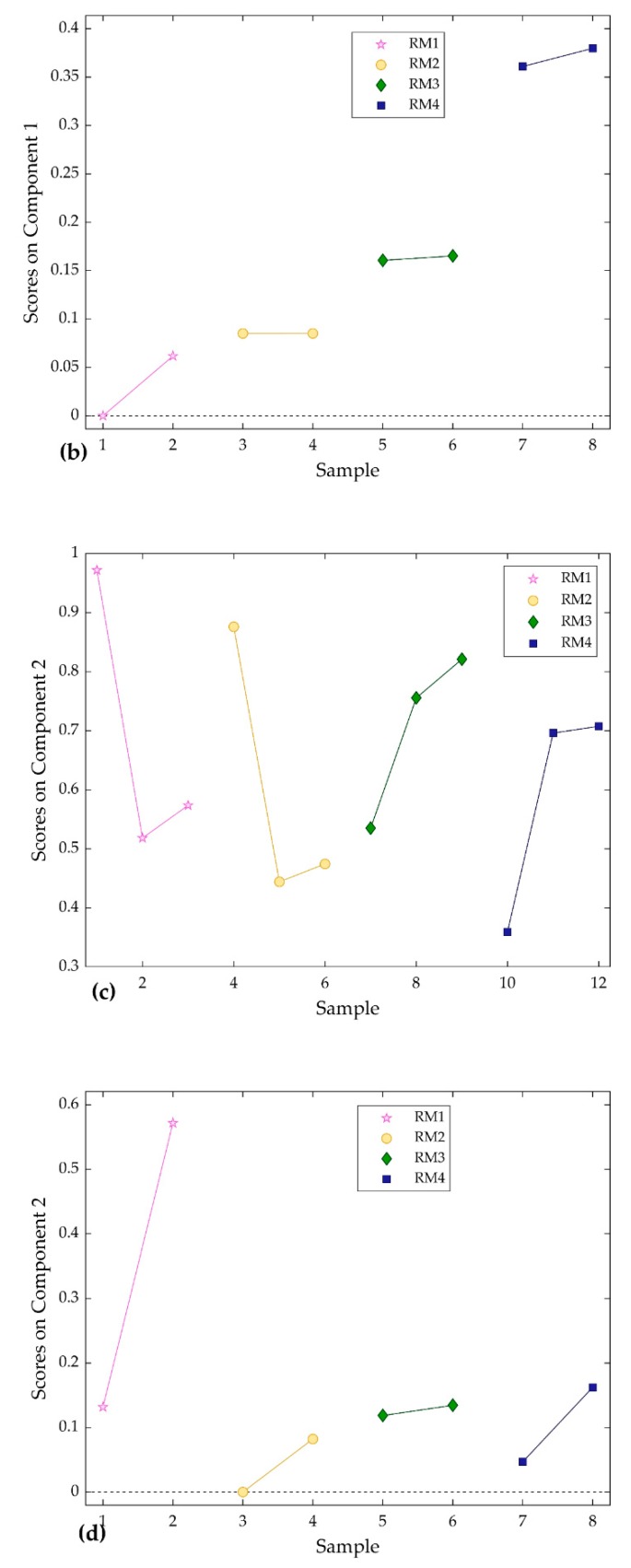

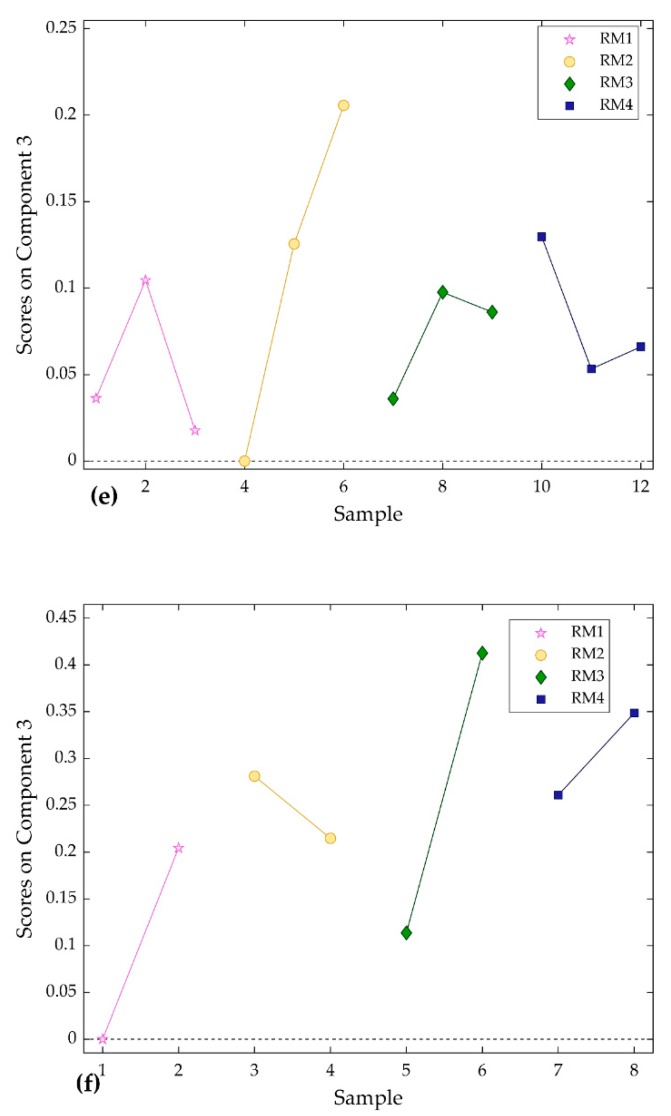
Scores of the two PARAFAC models with three components. **(a**) Scores of component 1 (TGIS), (**b**) scores of component 1 (TGO), (**c**) scores of component 2 (TGIS), (**d**) scores of component 2 (TGO), (**e**) scores of component 3 (TGIS), and (**f**) scores of component 3 (TGO).

Sample scores: Figure 9 shows scores of the components which indicate the relative quantity of each component in each sample. Scores of component 1 (Figure 9a,b) show the systematic relation between score values and increased RM in both TGIS and TGO. Score values increased with increased RM quantity. Scores of the second component are shown in Figure 9c,d. Relative quantity of component 2 decreased with increased RM quantity in both TGIS and TGO. Scores of the third component are shown in Figure 9e,f. No systematic relation is observed between increase in amount of RM and variation in score values in both TGIS and TGO.

**Figure 10:**
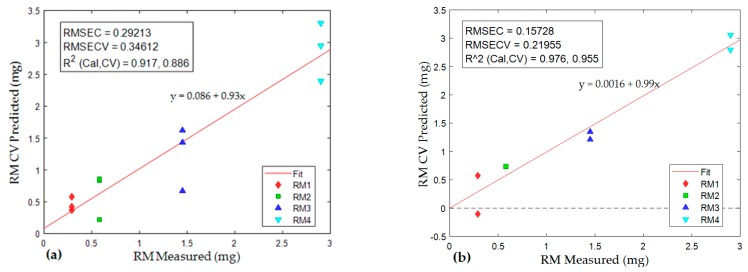
Partial least squares (PLS) linear regression of score values of samples in TGIS and TGO against measured values of RM. (**a**) Regression of TGIS samples, (**b**) regression of TGO samples. X-axis represents measured RM, Y-axis represents predicted RM. In the regression equation, y = predicted rubber materials (RM), x = measured rubber materials (RM).

Quantity estimation: following assessment of the score values, the component which showed increased score with increased RM were analyzed by PLS linear regression. Unknown quantity of component 1 was predicted for each sample (Figure 10) using score values of the component from PARAFAC models and measured values of RM (Table 1). The regression analysis showed R^2^ = 0.92 and 0.98, and RMSECV (Equation (2)) = 0.36 and 0.22 mg for TGIS and TGO respectively (Figure 10a,b).

## 4. Discussion

### 4.1. Decomposition of Overlying Components

According to different thermogravimetric analysis [5,14,17], rubber and tires degrade at pyrolysis temperature range of 340–550 °C. The wide degradation temperature range (20–650 °C) observed in this experiment, indicates the presence of more chemical substances than only rubber materials in tire granules (RM) in the evolved gases. The presence of multiple peaks in the landscape surfaces of the FTIR data of the evolved gases also points to the existence of overlying components.

The PARAFAC models resolved the components in this experiment. The models demonstrated good performance in capturing 89.5% and 90% of explained variations in tire granules in formulated sediment (TGIS) samples and tire granules only (TGO) samples respectively. A very high core consistency and small difference between components’ fit and unique fit means that PARAFAC model of TGO is highly stable. Models with similar performance [22,33] are considered to be unique and appropriate. The PARAFAC model of TGIS demonstrated similar performance with the exception of model unique fit diagnostics, which showed significant data points sharing between component 2 and component 3. Little or no difference between model fit and unique fit of component 1 in both models indicates the component being resolved uniquely. Decomposing the FTIR data with good diagnostic values conforms to the basic assumptions of the PARAFAC model. However, the models violate the non-covariability assumption because of the appearance of two substances with identical wavenumber cm^−1^ loadings in components 2 and 3 in both TGIS and TGO. These two substances are identified together likely due to generation of two substances that vary at similar intensities during the heating process (this point is discussed in the next section). The covariability phenomenon may have also happened to the rubber materials with similar spectral properties like natural rubber and synthetic rubber polymers present in tires [2,5,6] and yet identified as one component in the model. Despite this limitation, the PARAFAC models demonstrated good performance in accounting for differences between substances having similar chemical properties by keeping components 2 and 3 apart because of their difference in degradation temperatures. As a result, the models successfully resolved the overlying components into groups of three substances, which are discussed in the next section.

### 4.2. Identification of Components

The great advantage of coupling STA to FTIR is that it provides both physical and chemical properties for concurrent interpretation [11]. It permits compound analysis for fingerprint identification. Application of PARAFAC enhances the identification and quantification of substances by decomposing overlying components [22]. In this experiment, the PARAFAC models generated loadings. The loadings were matched against established fingerprint markers from Table 1 and Table 2 for assessment of chemical and physical properties of the three different components, which are discussed below.

Component 1: mode 2 loadings of the PARAFAC models in this experiment represent spectra of components, which reveal the fingerprint markers of substances. The mode 2 loadings of component 1 of both TGIS and TGO matched the identification marker wavenumbers cm^−1^ of rubber materials in tire (Table 2). Wavenumber cm^−1^ 2925, 2850, 1450, and 1377 are markers for ethylene propylene diene monomer (EPDM) while 960 and 890 are fingerprint markers for styrene butadiene rubber and butadiene in natural rubber respectively. Studies on FTIR spectra and rubber properties by [35,36] found similar wavenumber cm^−1^ peaks to identify natural and synthetic rubber.

Mode 3 loadings of the PARAFAC models provide information on the degradation temperature of components. The temperature loadings of component 1 shows a peak around 370 °C and shows a maximum peak at 450 °C in both TGIS and TGO. These temperature peaks are attributable to rubber material degradation (Table 3). Different studies have established a similar temperature range for rubber degradation. For example, the application note of Cambridge Polymer Group [17] states degradation temperature range of 340–550 °C with maximum degradation rate at 370 °C for natural rubber and 463 °C for styrene butadiene rubber. A study by [14] found tire degradation temperature in the range of 300–500 °C with maximum at 350 °C for natural rubber and 420 °C for styrene butadiene rubber. A similar temperature range (376.9–476.9 °C) is established by [5].

Component 2: assessment of component 2 showed no match with wavenumber cm^−1^ of tire materials in Table 2. The component in both models showed identification spectral peaks around wavenumber cm^−1^ 3735, 3560, and 1510 which are attributable to water in gaseous phase. It showed additional high peaks on the same spectra at 2350 and 670. These frequencies according to [37] are fingerprint markers to water and carbon dioxide substances.

Temperature loadings of component 2, peaks around 350 and 490 °C in TGIS. These peaks are closer to the of the three weight loss peaks observed in Figure 2. Similar temperature ranges are found to be typical for pyrolysis degradation of cellulose and lignin by [38,39] indicating component 2 stemmed mainly from plant material in TGIS. The corresponding component in TGO with no specific peak indicates the water vapor was released from the degradation of substances in tire granules.

Component 3: the wavenumber cm^−1^ of component 3 is similar to the wavenumber cm^−1^ of component 2 indicating water in gaseous phase in both TGIS and TGO albeit with low intensity at 2350 cm^−1^. The temperature loadings of component 3 increase continuously with temperature in both TGIS and TGO. This component is separated from component 2—which has wavenumber cm^−1^—due to its temperature loadings pattern. It is plausible therefore to suggest that component 3 is an artifact generated in the STA instrument during heating process rather than coming from the samples. The presence of data noise displayed by jagged loadings (components 2 and 3) and the possibility of condensation at sub-ambient temperature in purging process [40] supports the suggestion that the component may be external to the samples.

Therefore, the PARAFAC models separated rubber materials (RM) from other substances (water and carbon dioxide) in the FTIR data and facilitated the detection of RM in TGIS and TGO by both chemical and thermal properties. The fingerprint markers indicated that natural rubber (butadiene) and synthetic rubber (styrene butadiene rubber and ethylene propylene diene monomer) constitutes the identified RM. Ethylene propylene diene monomer (EPDM), a component of a tire sidewall, is an important marker in the identification. However, natural rubber (NR) and styrene butadiene rubber (SBR) could be the defining rubber materials for tread wear particles in the environment where the particles originate mainly from the tire thread.

### 4.3. Estimation of Quantity of Components

Quantifying tread wear particles through chemical analysis, particularly the use of FTIR, is not well established. Some [41] suggested FTIR as an alternative to be tested; while other studies [11] reported the inapplicability of FTIR in tire wear particles identification because of overlying components. However—in the PARAFAC model—if a component is identified as a specific chemical analyte, its quantity can be determined by adding a known quantity of the same chemical analyte—following Beer-Lambert’s law [22]. The rubber material component in this model represents all rubber materials in tire granules (RM). Calibrating the scores of the component identified as RM in TGIS and TGO against measured RM resulted in strong correlation between measured and predicted values in both TGIS and TGO. The goodness-of-fit test showed RMSECV of 0.36 and 0.22 mg in TGIS and TGO respectively. The regression analysis showed that predicted RM quantity were comparable to measured values in both TGIS and TGO indicating the possibility of estimating unknown quantity of RM by adding a known quantity of TGO.

Given a RM content of tire granules of 58%, estimation of quantity of tire granules is straightforward. As a result, the method effectively estimated quantity of tire particles from formulated sediments. The method can be applied to environmental samples to detect and quantify tread wear particles from soil and sediment. This offers environmental regulators and road authoritiesa new method that avoids extensive extraction and pretreatment steps common in microplastics analysis. With further development and attachment of PARAFAC to the FTIR analysis, the method can be automated for faster analysis of tread wear particles in soil and sediment samples.

## 5. Conclusions

This study introduced and demonstrated a new method to detect and eventually estimate quantity of tire particles in formulated sediments. The method consists of two steps, first utilizing a combination of instruments (STA and FTIR) to generate the data matrices, and then utilizes PARAFAC for the data analysis. In the current study, the STA and FTIR provided suitable data for the PARAFAC, which successfully decomposed the overlying components into different components, including the rubber materials and other compounds as well as artifacts. Otherwise, this would have been a challenge under conventional analytical methods. The prediction accuracy of the method may be improved with a laboratory designed to analyze rubber materials. A higher resolution STA instrument, which generates gases based on the degradation rate of substances and reduces artifacts, may also improve the prediction accuracy. 

## Figures and Tables

**Table 1:** Sample type, class, mass, rubber material quantity, number of replicates, and identification number. Sample type: TGIS = Tire granules in sediment, TGO = Tire granule only. Rubber materials (RM) = rubber materials: RM1 = samples with rubber material concentration of 0.29 mg, RM2 = samples with rubber material concentration of 0.58 mg, RM3 = samples with rubber material concentration of 1.45 mg, RM4 = samples with rubber material concentration of 2.90 mg.

RM	RM Quantity (mg)	Sample Type	Sample Mass (mg)	Number of Replicates	Identification Number
RM1	0.29	TGIS	50	3	1, 2, 3
		TGO	0.5	2	1, 2
RM2	0.58	TGIS	50	3	4, 5, 6
		TGO	1	2	3, 4
RM3	1.45	TGIS	50	3	7, 8, 9
		TGO	2.5	2	5, 6
RM4	2.90	TGIS	50	3	10, 11, 12
		TGO	5	2	7, 8

**Table 2:** Tire rubber material identification wavenumber cm^−1^. Polymers: NR = Natural Rubber, SBR = Styrene Butadiene Rubber, EPDM = Ethylene Propylene Diene Monomer.

Polymer	Functional Group	Wavenumber cm^−1^	Reference
NR, SBR, EPDM	Methyl	2964	Gunasekaran, Natarajan, and Kala, [18]
NR	Methylene	2950, 2853	Gunasekaran, Natarajan, and Kala, [18]
NR, SBR, EPDM	OH	3470	Gunasekaran, Natarajan, and Kala, [18]
EPDM		1076	V. M. LiPtvinow and P. P. De, [11]
SBR	Polystyrene	750, 700	Fernández-Berridi et al. and Gunasekaran, Natarajan, and Kala, [14,18]
EPDM	Propylene	1461, 1376	Gunasekaran, Natarajan, and Kala, (2007) [18]
SBR	Vinyl	990, 910	Fernández-Berridi et al. and Gunasekaran, Natarajan, and Kala, [14,18]
SBR	Butadiene	960	Gunasekaran, Natarajan, and Kala, [18]
NR, EPDM	Ethylene	722, 815	Fernández-Berridi et al. [14]
EPDM, (NR)	Alkyl	2929, 2856	Fernández-Berridi et al. and Gunasekaran, Natarajan, and Kala, [14,18]
NBR	Alkyl	2230	Gunasekaran, Natarajan, and Kala, [18]

**Table 3:** Rubber materials pyrolysis degradation temperature in °C. Polymers: NR = Natural Rubber, SBR = Styrene Butadiene Rubber, EPDM = Ethylene Propylene Diene Monomer.

Polymer	Temperature (°C)	Reference
NR, SBR, EPDM	376−476, 400−550	Januszewicz, Klein, Klugmann-Radziemska, and Kardas, and M. Engineering and S. Republi, [5,34]
NR	350	Fernández-Berridi et al. [14]
NR, SBR, EPDM	300−500	Fernández-Berridi et al. [14]
SBR	424	Fernández-Berridi et al. [14]
